# The Role of Prostaglandins and COX-Enzymes in Chondrogenic Differentiation of ATDC5 Progenitor Cells

**DOI:** 10.1371/journal.pone.0153162

**Published:** 2016-04-06

**Authors:** Marjolein M. J. Caron, Pieter J. Emans, Kathleen Sanen, Don A. M. Surtel, Andy Cremers, Daan Ophelders, Lodewijk W. van Rhijn, Tim J. M. Welting

**Affiliations:** Department of Orthopaedic Surgery, Caphri School for Public Health and Primary Care, Maastricht University Medical Centre, Maastricht, the Netherlands; Boston University Goldman School of Dental Medicine, UNITED STATES

## Abstract

**Objectives:**

NSAIDs are used to relieve pain and decrease inflammation by inhibition of cyclooxygenase (COX)-catalyzed prostaglandin (PG) synthesis. PGs are fatty acid mediators involved in cartilage homeostasis, however the action of their synthesizing COX-enzymes in cartilage differentiation is not well understood. In this study we hypothesized that COX-1 and COX-2 have differential roles in chondrogenic differentiation.

**Methods:**

ATDC5 cells were differentiated in the presence of COX-1 (SC-560, Mofezolac) or COX-2 (NS398, Celecoxib) specific inhibitors. Specificity of the NSAIDs and inhibition of specific prostaglandin levels were determined by EIA. Prostaglandins were added during the differentiation process. Chondrogenic outcome was determined by gene- and protein expression analyses.

**Results:**

Inhibition of COX-1 prevented Col2a1 and Col10a1 expression. Inhibition of COX-2 resulted in decreased Col10a1 expression, while Col2a1 remained unaffected. To explain this difference expression patterns of both COX-enzymes as well as specific prostaglandin concentrations were determined. Both COX-enzymes are upregulated during late chondrogenic differentiation, whereas only COX-2 is briefly expressed also early in differentiation. PGD_2_ and PGE_2_ followed the COX-2 expression pattern, whereas PGF_2α_ and TXA_2_ levels remained low. Furthermore, COX inhibition resulted in decreased levels of all tested PGs, except for PGD_2_ and PGF_2α_ in the COX-1 inhibited condition. Addition of PGE_2_ and PGF_2α_ resulted in increased expression of chondrogenic markers, whereas TXA_2_ increased expression of hypertrophic markers.

**Conclusions:**

Our findings point towards a differential role for COX-enzymes and PG-production in chondrogenic differentiation of ATDC5 cells. Ongoing research is focusing on further elucidating the functional partition of cyclooxygenases and specific prostaglandin production.

## Introduction

The chondrogenic differentiation process is accompanied by a stage-dependent expression of important chondrogenic markers: Sox9 (SRY (sex determining region Y)-box 9) is a primary determinant of chondrogenic differentiation from early differentiation stage onwards [1, 2], while its transcriptional targets; collagen type 2 (Col2a1) and aggrecan (Acan) are prominently expressed by more mature, extracellular matrix (ECM) producing chondrocytes. On the other hand, Collagen type 10 (Col10a1) and its transcription factor Runx2 (Runt-related transcription factor 2) are specifically expressed by hypertrophic differentiating chondrocytes [1, 2]. Cyclooxygenases, also known as prostaglandin H synthases, are enzymes that catalyse the conversion of arachidonic acid to prostaglandin H_2_, a rate-limiting step in the generation of prostaglandins [3, 4]. Prostaglandin H_2_ (PGH_2_) is converted by specific synthases to the main prostaglandin subgroups PGD_2_, PGE_2_, PGI_2_, PGF_2α_ and thromboxane A2 (TXA_2_), which can be converted into various other intermediates [5, 6]. To date three COX isoforms have been described: COX-1, COX-2 and COX-3. The first two are the most prevalent and best described isoforms [7]. The COX-3 isoform is a splice variant of COX-1; however there is much debate on the function of COX-3 [8–10]. Both COX-1 and COX-2 isoforms catalyse the same enzymatic reactions and are structurally related [11]. They have remarkable differences regarding their tissue distribution, expression levels and their ability to respond to various stimuli [7, 12, 13]. COX-1 is constitutively expressed by most mammalian cells and regarded as the “housekeeping” cyclooxygenase. On the other hand, COX-2 expression is low in most tissues but can be rapidly induced upon exposure to various stimuli such as inflammation, mechanical stress and injury [3, 7] due to inducible enhancer elements in its promoter [11].

Studies investigating the roles of cyclooxygenases and the effects of prostaglandins on (*in vitro*) chondrogenic differentiation have mainly focussed on COX-2 (inhibition) and PGE_2_ [14–20]. However, the contribution of COX-1 and other prostaglandins in chondrogenic differentiation is largely ignored. Given their different roles in cellular prostaglandin synthesis, it is not unlikely that the two COX enzymes are differently involved in determining the outcome of the chondrogenic differentiation process.

We have previously shown that COX-2 expression is transiently upregulated during the first hours in chondrogenic differentiation of progenitor cells [21] and a second time later in differentiation, when its expression coincidences with chondrocyte extracellular matrix synthesis [22]. In addition, we found that inhibition of COX-2 enzymatic activity results in a reduction of the hypertrophy outcome of the chondrogenic differentiation process [22]. However, whether this observation is specific for COX-2 and how this relates to COX-1 activity and specific prostaglandin involvement is unclear.

We hypothesized that COX-1 and COX-2 have differential roles in the chondrogenic differentiation process of progenitor cells and that their specific inhibition will thereby differently influence the outcome of the differentiation process. The characterisation of the function of the COX-enzymes and prostaglandins holds the promise to gain a deeper understanding of the chondrogenic differentiation process and thereby may uncover novel chances to influence cartilage tissue regeneration.

## Methods

### Immunohistochemistry

Growth plates were isolated from tibias of 6 weeks old C57BL/6 mice (approved by the local Animal Ethics Committee according to Dutch law; MUMC DEC approval 2008–042). Mice were euthanized using O2/CO2 asphyxiation. The tibias were fixed in formalin and decalcified in 0.5 M EDTA (Ethylenediaminetetraacetic acid) pH 7.8. Tibias were dehydrated, embedded in paraffin and five micrometer sections were cut. Sections were deparaffinized and antigen retrieval for COX-1 sections was performed by digestion with 0.4% hyaluronidase (Sigma-Aldrich, St. Louis, MO, USA) for 30 minutes at 37°C. For COX-2 detection, sections were incubated in hot citrate buffer for 30 minutes. Endogenous peroxidase activity was inactivated and sections were blocked with 10% normal sheep serum. Primary antibodies (anti-COX-1 (160110, Cayman Chemical, Ann Arbor, USA) and anti-COX-2 (610203, BD Transduction Laboratories)) were used in 1:100 dilution in PBS (Phosphate buffered saline). After washing in PBS-Tween20, bound antibodies were detected with HRP (horseradish peroxidase)-labelled secondary antibodies (Dako, EnVision+ System-HRP labelled Polymer). Bound secondary antibodies were visualized using DAB substrate (Dako, Glostrup, Denmark). Stained sections were counterstained with Mayer’s Hematoxylin (Dako), dehydrated and mounted in Histomount (Thermo Shandon).

### ATDC5 culture

ATDC5 cells [23] (RIKEN Cell Bank, Japan) were cultured in a humidified atmosphere at 37°C, 5% CO_2_. Proliferation medium consisted of DMEM (Dulbecco’s modified eagle medium)/F12 (Life Technologies, Bleiswijk, the Netherlands), 5% FCS (fetal calf serum) (Lonza, Basel, Switzerland), 1% antibiotic/antimycotic (Penicillin/Streptomycin; Life Technologies) and 1% NEAA (non-essential amino acids; Life Technologies). Differentiation medium comprised proliferation medium supplemented with 10 μg/ml insulin (Sigma-Aldrich), 10 μg/ml transferrin (Roche Applied Science, Indianapolis, IN, USA) and 30 nM sodium selenite (Sigma-Aldrich). Cells were plated at 6400 cells/cm^2^. Differentiation medium was changed every two days (day 0–10) and daily (from day 10 onwards). To inhibit COX-1 activity SC-560 (1 μM; Cayman Chemical, Ann Arbor, USA) or Mofezolac (5 μM; Sigma-Aldrich) were used and applied during the differentiation process. Similarly, COX-2 activity was inhibited by NS398 (20 μM; Cayman Chemical) or Celecoxib (10 μM; LC laboratories). PGD_2_, PGE_2_, PGF_2α_ and U-46619 (a TXA_2_ receptor agonist, herein referred to as TXA_2_) (Cayman Chemical) were applied daily during the differentiation process at 0, 0.1, 1 or 10 μM concentrations. These concentrations were selected in the range of the highest concentrations reported in the culture supernatants of control differentiated t = 14 days ATDC5 cells. PGI_2_ (from the same manufacturer) was not applied in these experiments, as it is unstable in culture medium and therefore could not be used in a reliable manner.

### Prostaglandin measurement

Prostaglandin concentration of PGD_2_, PGE_2_, PGF_2α_ and TXA_2_ was determined in culture supernatants using ELISA’s according to the manufacturers’ protocol (Cayman Chemical). PGI_2_ concentrations could not be determined in a reliable manner by the ELISA’s from the same manufacturer and was therefore not included in these experiments.

### COX specificity assay

The colorimetric COX inhibitor screening assay kit (Cayman Chemical) was used according to the manufacturers’ protocol to determine COX isoform-specific inhibitory capacity for SC-560, Mofezolac, NS398 and Celecoxib.

### RT-qPCR

For RNA isolation, cells were disrupted with 300 μl Trizol (Life Technologies). RNA isolation, RNA quantification by UV-spectrometry (Nanodrop, Thermo Scientific), and cDNA synthesis were performed as described before [21, 22]. Real time quantitative PCR (RT-qPCR) was performed using Mesagreen qPCR mastermix plus for SYBR^®^ Green (Eurogentec, Seraing, Belgium). A CFX96 Real-Time PCR Detection system (Biorad, Hercules, CA, USA) was used for amplification: initial denaturation at 95°C for 10 minutes, followed by 40 cycles of DNA amplification (denaturing for 15 seconds at 95°C and annealing for 1 minute at 60°C) followed by a dissociation curve. Data were analysed using the standard curve method, mRNA expression was normalized to a reference gene (β-actin) and gene expression was calculated as fold change compared to day 0 (t = 0 in graphs). Validated primer sequences are depicted in Table 1.

**Table 1:** Primer sequences for RT-qPCR.

Oligo sets	Forward	Reverse
Collagen type 2 (Col2a1)	‘5-TGGGTGTTCTATTTATTTATTGTCTTCCT-3’	‘5-GCGTTGGACTCACACCAGTTAGT-3’
Collagen type 10 (Col10a1)	‘5-CATGCCTGATGGCTTCATAAA-3’	‘5-AAGCAGACACGGGCATACCT-3’
SRY (sex determining region Y)-box 9 (Sox9)	‘5-AGTACCCGCACCTGCACAAC-3’	‘5-TACTTGTAGTCCGGGTGGTCTTTC-3’
Runt-related transcription factor 2 (Runx2)	‘5-CGATGAAGACCCCAACCCTAA-3’	‘5-ACTGGTAATGGCATCAAGGGATA-3’
Cyclooxygenase 1 (COX-1)	‘5-TCCTCACAGTGCGGTCCAA-3’	‘5-AAGGCCTCCCAGCTGATGTAG-3’
Cyclooxygenase 2 (COX-2)	‘5-GGCCATGGAGTGGACTTAAA-3’	‘5-AAGTGGTAACCGCTCAGGTG-3’
β-Actin	‘5-ACAGGATGCAGAAGGAGATTACTG-3’	‘5-CCACCGATCCACACAGAGTACTT-3’

The 5*’*– 3*’* forward and reverse oligonucleotide sequences used for RT-qPCR are listed

### Immunoblotting

Cells were washed with 0.9% NaCl and lysed in RIPA buffer. Extracts were sonicated on ice using the Soniprep 150 (MSE, London, UK) at amplitude 10 for 14 cycles (1 second sonication and 1 second pause). Insoluble material was removed by centrifugation (13,000 x g, 4°C). Protein concentration was determined using the BCA protein assay (Sigma-Aldrich). Polypeptides were separated by SDS-PAGE (sodium dodecyl sulfate polyacrylamide gel electrophoresis; samples were equally loaded) and transferred to nitrocellulose membranes by electroblotting. Primary antibodies (all 1:100 dilution) for immunodetection were polyclonal goat anti-Col2a1 (1320–01, Southern Biotech, Birmingham, AL, USA), polyclonal rabbit anti-Col10a1 (234196, Calbiochem, Darmstadt, Germany), polyclonal rabbit anti-Sox9 (ab 3697, Abcam), mouse monoclonal anti-Runx2 (D130-3, MBL, Woburn, USA) and mouse monoclonal anti-α-Tubulin (T6074, Sigma-Aldrich; 1:10.000 dilution). Bound primary antibodies were detected with secondary immunoglobulins conjugated with horseradish peroxidase (Dako) and visualized by enhanced chemiluminescence (ECL). ECL signals were quantified using ImageJ 1.46f software, and relative differences, corrected for background and housekeeper, were determined as compared to control conditions.

### GAG content

Cells were washed with 0.9% NaCl and fixed with 4% paraformaldehyde. GAG deposition was detected by alcian blue staining. Fixed cells were incubated overnight with 1% alcian blue (Acros Organics, Geel, Belgium) in 0.1 M HCl. Alcian blue was extracted from the cells by incubation with guanidine-HCl (6 M) for 2 hours under continuous agitation. Extracted alcian blue was determined spectrophotometrically at 645 nm using a plate reader (Biorad).

### Statistics

In the figures bars represent average value of 3 individual experiments (performed in triplicate) and error bars represent mean ± standard error of mean (s.e.m.). Statistical significance (p <0.05) was determined by ANOVA with Bonferroni *post hoc* analysis using GraphPad PRISM 5.0 (La Jolla, CA, USA).

## Results

### Expression of COX-1 and COX-2 during chondrogenic differentiation

**Fig 1:**
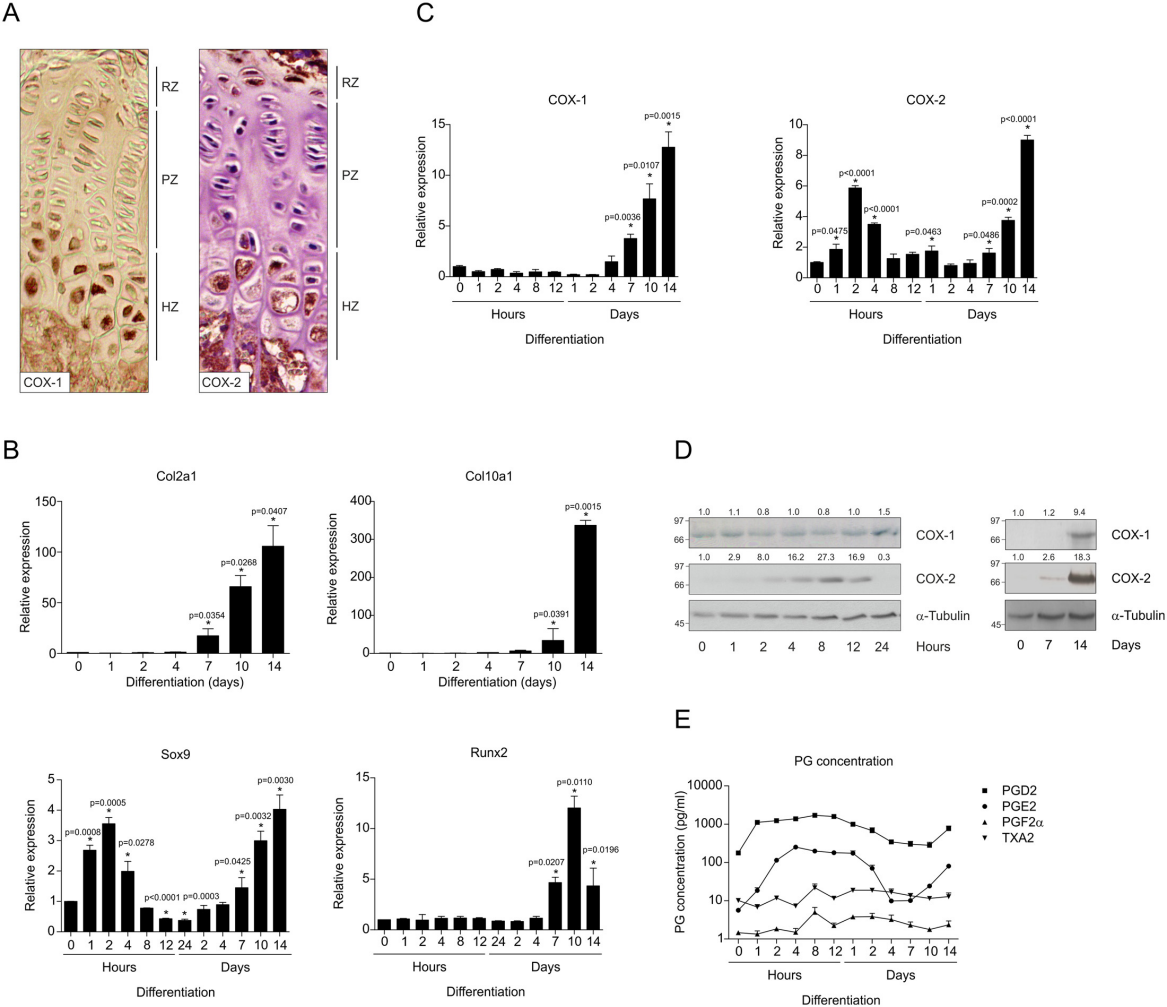
Expression of cyclooxygenases during chondrogenic differentiation from progenitor cells. **A:** Expression of COX-1 and COX-2 in growth plates was determined by IHC. RZ = resting zone, PZ = proliferative zone, HZ = hypertrophic zone. **B:** Col2a1, Col10a1, Sox9 and Runx2 mRNA expression during chondrogenic differentiation of ATDC5 cells. **C:** COX-1 and COX-2 mRNA expression. In graphs, error bars represent mean ± s.e.m.(standard error of the mean; n = 3). * indicates *p* < 0.05. **D:** Protein expression of COX-1 and COX-2 in differentiation. Molecular weight markers (kDa) are depicted on the left of immunoblots and relative quantifications are depicted on top of immunoblots. **E:** Medium concentrations of PGD_2_, PGE_2_, PGF_2α_ and TXA_2_ during chondrogenic differentiation.

To examine the involvement of COX-1 and COX-2 during chondrogenic differentiation, spatiotemporal expression of these enzymes was first determined in murine growth plates. Expression of COX-1 and COX-2 was specifically detected in chondrocytes located in the hypertrophic zone of the growth plate, whereas COX-2 was also detected in cells in the resting zone (Fig 1A). To verify these results, we analysed expression of COX-1 and COX-2 during chondrogenic differentiation of ATDC5 cells. ATDC5 differentiation follows a well-defined and -established endochondral program from undifferentiated chondroprogenitor to hypertrophic chondrocyte [23–25]. Col2a1 and Sox9 were increasingly expressed from day 7 onwards in ATDC5 differentiation, establishing chondrogenic differentiation of ATDC5 cells. Moreover, Sox9 expression was also increased early in differentiation (first hours) (Fig 1B). Expression of chondrocyte hypertrophic genes Runx2 and Col10a1 increased from day 7 and day 10 onwards, respectively (Fig 1B). COX-1 expression was increased in ATDC5 differentiation from day 7 onwards (Fig 1C/1D), correlating with the expression timing of Col2a1 and Col10a1 (Fig 1B). COX-2 expression was transiently upregulated in the first hours of chondrogenic differentiation and increased again from day 7 onwards (Fig 1C/1D). These data correlate with the growth plate expression patterns of COX-1 and COX-2 as well as with our previous findings [21, 22]. To functionally determine COX enzyme activity during ATDC5 chondrogenic differentiation the concentration of the prostaglandins PGD_2_, PGE_2_, PGF_2α_ and TXA_2_ was determined in culture supernatants. Fig 1E shows that PGD_2_ and PGE_2_ concentrations in the culture supernatant changed throughout differentiation; during the first hours/days of differentiation levels quickly raised and decreased again, to increase after about a week again, resembling the bi-phasic expression dynamics of COX-2. PGF_2α_ and TXA_2_ levels remained stably low during ATDC5 chondrogenic differentiation.

### COX-1 and COX-2 inhibition differentially influence ATDC5 chondrogenic outcome and specific prostaglandin concentrations

**Fig 2:**
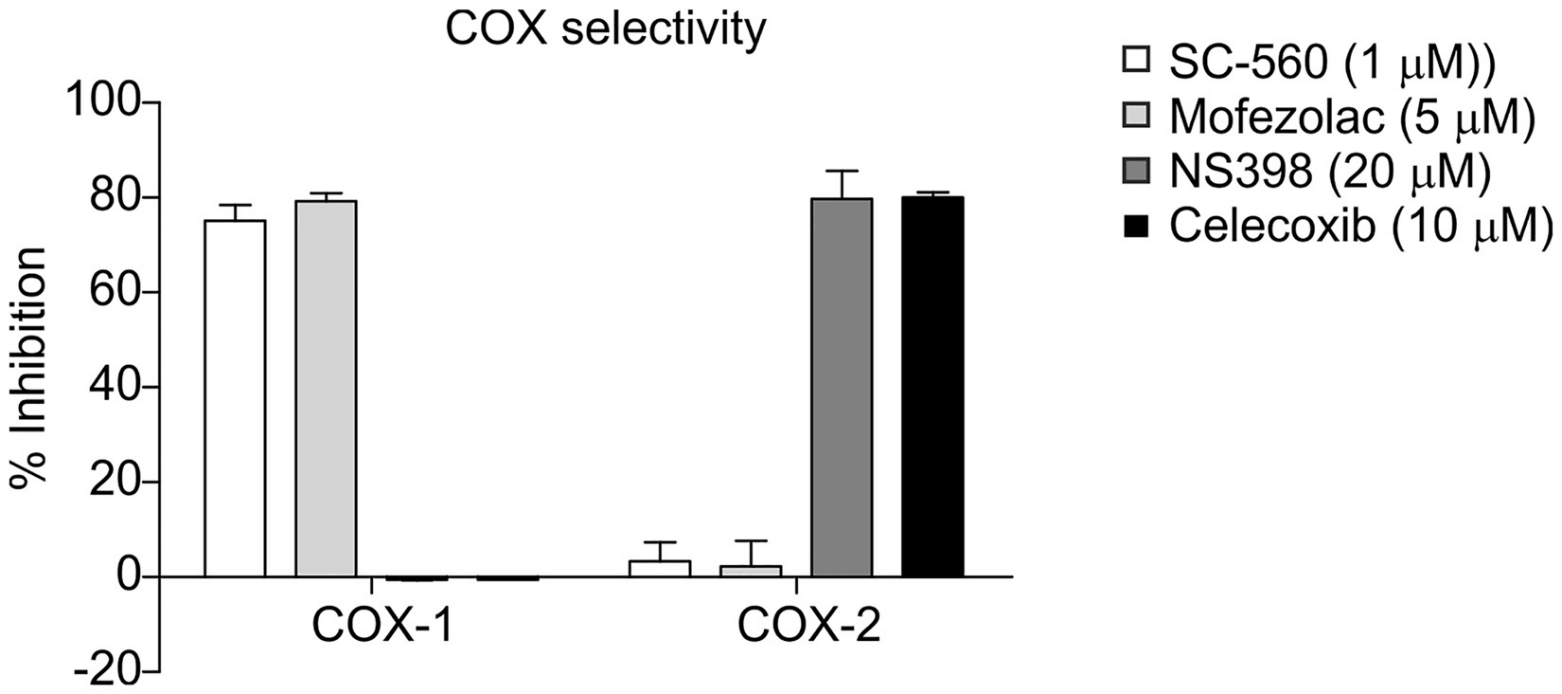
Specificity of COX-1 and COX-2 inhibitors. The selectivity of SC-560 and Mofezolac for COX-1 over COX-2 and selectivity of NS398 and Celecoxib for COX-2 over COX-1 was determined using a COX-1/COX-2 colorimetric inhibition assay. The inhibitory capacity was determined as “% inhibition” compared to a maximum activity control condition. Bars represent average inhibition percentages of COX-1 and COX-2. Measurements were done in triplicate and error bars represent the variances in inhibitory activity.

Inhibitor concentrations at which specificity of the inhibitor for COX-2 over COX-1, or COX-1 over COX-2 was highest were determined, and revealed optimal inhibitor concentrations for which similar inhibition capacities between the inhibitors were achieved, combined with the highest COX isoform specificity. For the COX-1 inhibitors these optimal concentrations were 1 μM for SC-560 and 5 μM for Mofezolac and for COX-2 inhibitors 20 μM for NS398 and 10 μM for Celecoxib (Fig 2).

**Fig 3:**
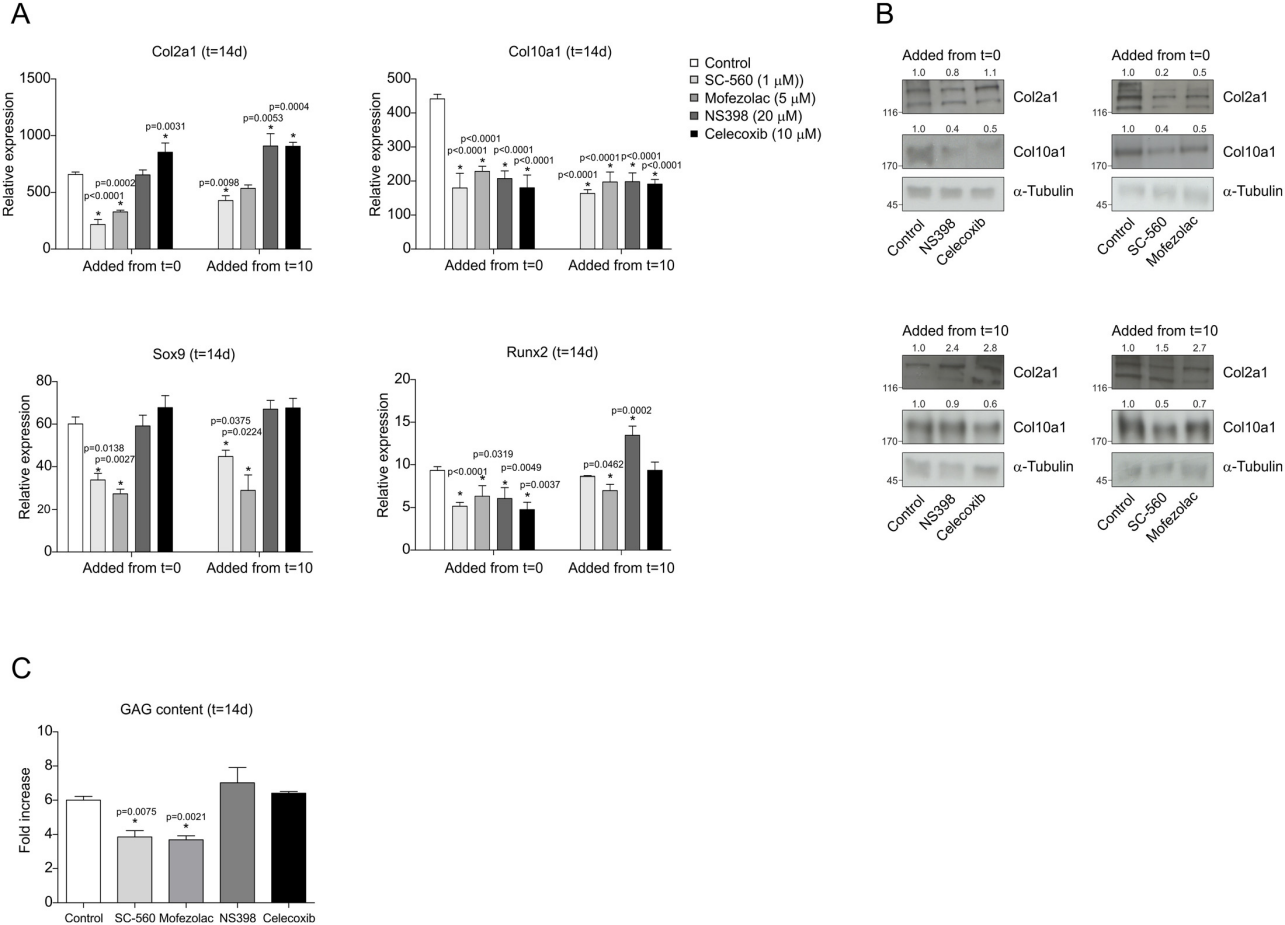
Effects of COX-1 and COX-2 specific inhibitors on chondrogenic differentiation of ATDC5 cells. **A:**. Col2a1, Col10a1, Sox9 and Runx2 mRNA expression was determined at day 14 in ATDC5 differentiation. **B:** Protein expression of Col2a1 and Col10a1 at day 14 in differentiation. Molecular weight markers (kDa) are depicted on the left of immunoblots and relative quantifications are depicted on top of immunoblots. **C:** Glycosaminoglycans (GAGs) were stained and fold change of t = 14 samples was calculated as compared to t = 0 samples. Fold change (DNA) from samples from t = 14 was calculated relatively to day 0. In graphs, error bars represent mean ±s.e.m. (n = 3). * indicates *p* < 0.05.

To determine how chondrogenic differentiation of ATDC5 cells responds to COX-1 *versus* COX-2 inhibition, the different isoform-specific COX inhibitors were added from the start of differentiation or from day 10 onwards (to determine whether a potential effect depends on the differentiation status of the chondrocyte). As compared to control conditions, inhibition of COX-1 from the start of differentiation resulted in decreased Col2a1, Col10a1, Sox9 and Runx2 mRNA and protein levels at day 14 in differentiation (Fig 3A/3B). Inhibition of COX-1 from day 10 onwards resulted in similar, although less pronounced effects on expression of these genes. Inhibition of COX-2 starting at day 1 or day 10, on the other hand, did not lead to decreased expression of Col2a1 and Sox9 at day 14 in differentiation and even increased Col2a1 expression (for Celecoxib at both time points and for NS398 when added from day 10 onwards in differentiation) (Fig 3A/3B). A similar trend was detected for Sox9, although not significant. Inhibition of COX-2 did however affect the expression of chondrocyte hypertrophy genes Runx2 and Col10a1 at day 14 in differentiation of ATDC5 cells, when COX-2 was inhibited from the start of the experiment [22]. Inhibition of COX-2 from day 10 onwards resulted in similar although less pronounced effects on these chondrocyte hypertrophy genes. GAG content was decreased due to COX-1 specific inhibition at day 14 in differentiation and no effect on GAG content was observed for the COX-2 specific inhibition (Fig 3C).

**Fig 4:**
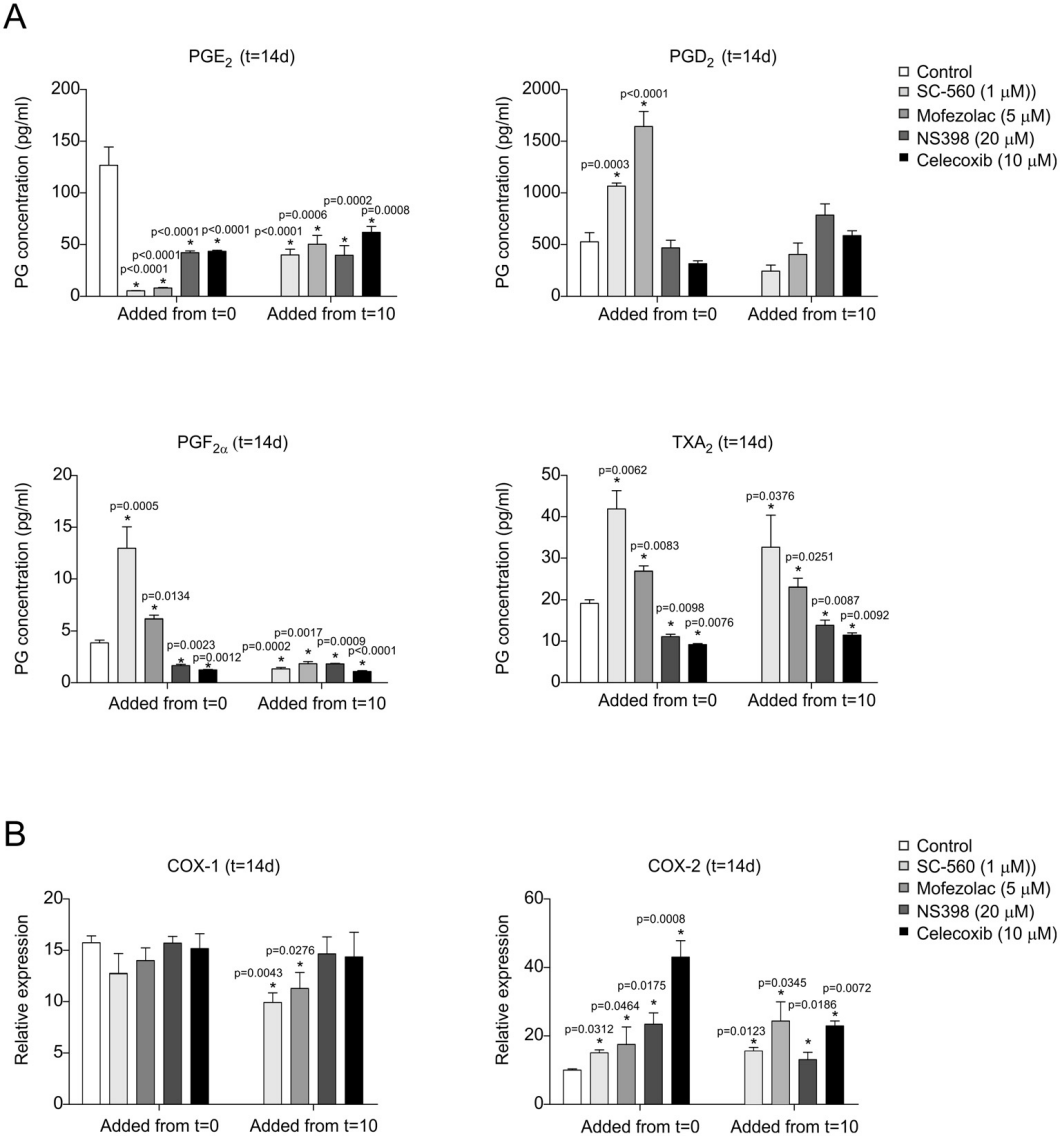
COX-1 and COX-2 specific inhibitors differentially influence prostaglandin levels during ATDC5 chondrogenic differentiation. Successful COX-1 or COX-2 inhibition by the inhibitors and possible differences in specific prostaglandin production was determined at day 14 in ATDC5 differentiation. NS398, Celecoxib, SC-50 or Mofezolac were added from the start of differentiation or from day 10 onwards. **A:** PGE_2_, PGD_2_, PGF_2α_ and TXA_2_ medium concentrations were determined at day 14 in differentiation by prostaglandin specific ELISA. **B:** COX-1 and COX-2 mRNA expression at day 14 in differentiation was determined by RT-qPCR (relative to t = 0 and corrected for β-actin). In graphs, error bars represent mean ± s.e.m. (n = 3). * indicates *p* < 0.05.

In order to confirm successful COX-1 or COX-2 inhibition during chondrogenic differentiation of ATDC5 cells and to determine whether both isoforms are equally involved in specific prostaglandin synthesis, PGE_2_, PGD_2_, PGF_2α_ and TXA_2_ levels were measured. As shown in Fig 4A (upper left panel), inhibition of COX-1 as well as COX-2 from day 0 or day 10 onwards caused a significant reduction in the PGE_2_ concentration at day 14 in differentiation, with the most efficient inhibition caused by both COX-1 inhibitors. COX-2 inhibition (both from day 0 and day 10 onward) by NS398 or Celecoxib also resulted in reduced PGF_2α_ and TXA_2_ levels, but had no significant effect on PGD_2_ levels. Interestingly, COX-1 inhibition from the start of differentiation in ATDC5 cells resulted in increased levels of PGD_2_, PGF_2α_ and TXA_2_ at day 14 in differentiation (Fig 4A). In contrast, COX-1 inhibition from day 10 in differentiation onwards, did not significantly influence PGD_2_ levels, but again increased TXA_2_ levels, while PGF_2α_ levels in this condition were decreased as compared to control. Although the inhibitors enzymatically interfere with COX-activity, potential feedback mechanisms responding to diminished COX-activity might influence the level of COX-1 and COX-2 expression. To address this possibility we measured expression of COX-1 and COX-2 mRNAs. Inhibition of COX-1 by SC-560 or Mofezolac from the start of differentiation did not influence COX-1 mRNA expression at day 14 in ATDC5 differentiation, however when COX-1 was inhibited from day 10 onwards a slight decrease in COX-1 mRNA expression was detected. Interestingly, COX-2 mRNA expression increased in all tested COX-1 inhibitory conditions (Fig 4B). In cells treated with COX-2 specific inhibitors, COX-2 mRNA expression itself was increased as well, whereas COX-1 mRNA expression remained unaltered.

### Addition of specific prostaglandins during chondrogenic differentiation of ATDC5 cells

**Fig 5:**
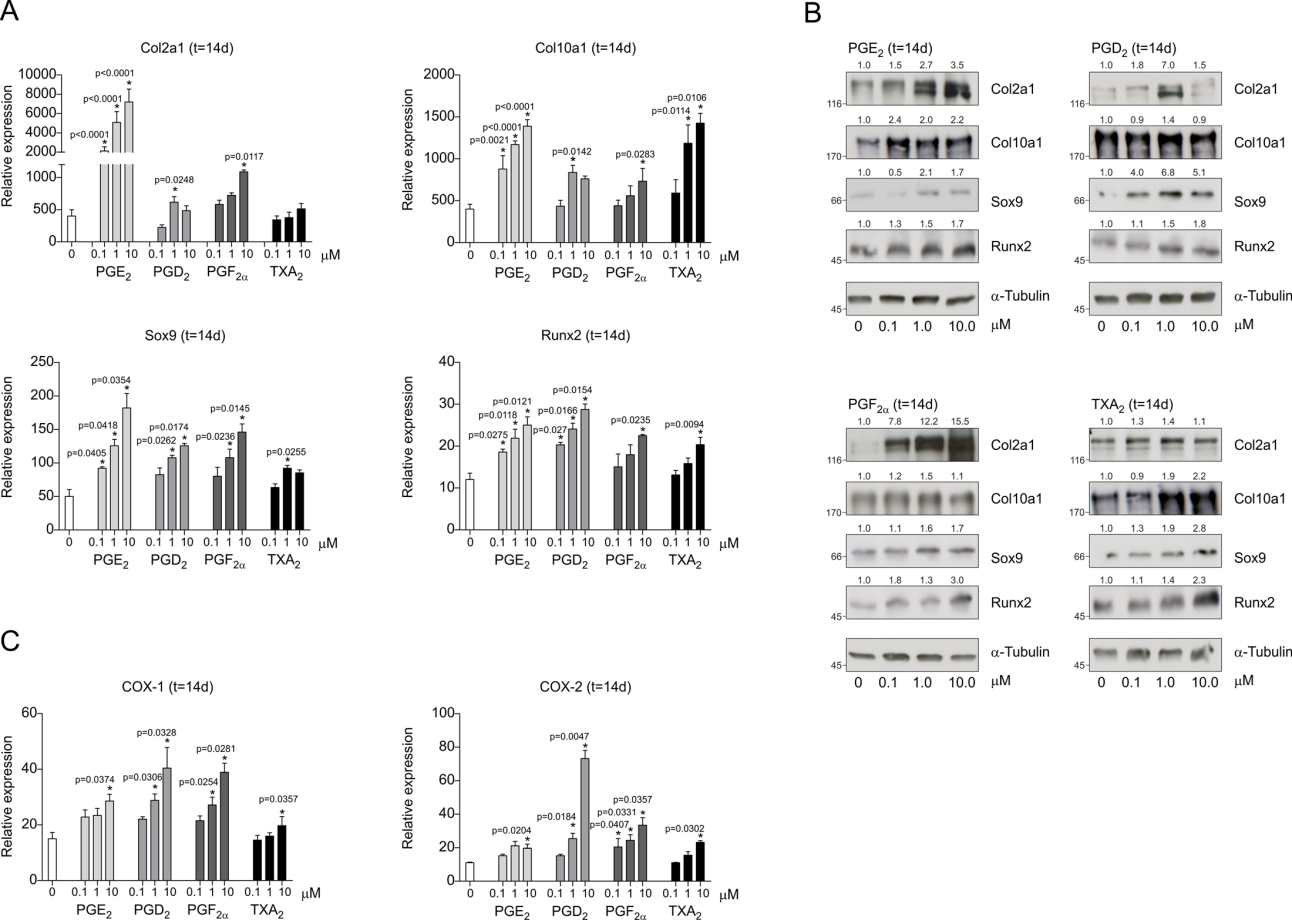
Prostaglandins differently regulate chondrogenic differentiation of ATDC5 cells. Increasing concentrations of PGE_2_, PGD_2_, PGF_2α_, TXA_2_ were added to differentiating ATDC5 cells. **A:** Col2a1, Col10a1, Sox9 and Runx2 mRNA expression was determined at day 14 in differentiation by RT-qPCR (relative to t = 0 and corrected for β-actin). **B:** Protein expression of Col2a1, Col10a1, Sox9 and Runx2 at day 14 in differentiation. Molecular weight markers (kDa) are depicted on the left of immunoblots and relative quantifications are depicted on top of immunoblots. **C:** In samples from (A) mRNA expression of COX-1 and COX-2 was determined. In graphs, error bars represent mean ± s.e.m. (n = 3). * indicates p < 0.05.

As inhibition of COX-1 or COX-2 has different consequences on the chondrogenic outcome and appears to affect downstream specific prostaglandin synthesis differently, we tested whether these prostaglandins differently influence chondrogenic differentiation. To this end, increasing concentrations of prostaglandins PGD_2_, PGE_2_, PGF_2α_ and TXA_2_ were added to differentiating ATDC5 cells. Addition of PGE_2_ and the highest concentration of PGF_2α_ caused an increased expression of Col2a1, Col10a1, Sox9 and Runx2 mRNA and protein at day 14 in differentiation (Fig 5A/5B). TXA_2_ addition specifically resulted in increased expression of the hypertrophic genes Col10a1 and Runx2 and had almost no effects on the expression of chondrogenic genes Col2a1 and Sox9. Expression of Sox9 and Runx2 was increased by addition of PGD_2_ but this did only result in an increased trend in Col2a1 and Col10a1 expression (Fig 5A/5B). Furthermore addition of the individual prostaglandins resulted in increased COX-1 and COX-2 mRNA expression (Fig 5C).

## Discussion

The majority of the studies reporting on the function of COX enzymes in cartilage and bone tissues focused on the function of COX-2 and PGE_2_ in mature chondrocytes and osteoblasts [14–20]. This leaves the role of COX-1 and other prostaglandins during chondrogenic differentiation from progenitor cells poorly studied.

We found that COX-1 and COX-2 presented differential temporospatial expression patterns in developing growth plates; both isoforms were abundantly expressed in the hypertrophic chondrocytes of the growth plate, whereas specifically COX-2 was additionally found to be expressed in resting zone cells. These findings are in part in contrast with previous findings reported by Brochhaussen *et al*. [14] who reported abundant expression of COX-1 throughout the rat growth plate and increased COX-2 expression in proliferative and hypertrophic zone of rat growth plates. Our results from murine growth plates showed interesting parallels with our *in vitro* ATDC5 observations though. Here COX-1 expression was only upregulated late in chondrogenic differentiation whereas COX-2 expression showed an initial peak during early differentiation and expression increased again from day 7 in differentiation and further. Recently, we and others have shown that NF-κB signalling is essential for early chondrogenic differentiation and that early NF-κB activation is required for the transient expression of COX-2 during early differentiation [21, 26]. Taken the above transient early COX-2 expression into account and the increased presence of COX-2 in the resting zone cells of murine growth plates, it is tempting to speculate on the role for COX-2 early in chondrogenic differentiation. However, based on expression of chondrogenic genes, we did not find any differences for COX-2 inhibition between day 0 and day 10.

The partially distinct expression patterns for COX-1 and COX-2 may suggest differential involvement of the two COX isoforms in chondrogenic differentiation of ATDC5 progenitor cells. When COX-1 was specifically inhibited an overall decrease was found in induction of chondrogenic and hypertrophic genes as Col2a1, Sox9, Runx2 and Col10a1. Furthermore, chondrogenic gene expression responded similar when COX-1 was specifically inhibited late in differentiation, implying that COX-1 activity serves a crucial role during various phases of chondrogenic differentiation. These findings are supported by literature also showing adverse effects on proteoglycan synthesis and content when COX-1 was inhibited [27]. Notably, in accordance with the overall chondrogenic inhibitory effect by specific COX-1 inhibitors, COX-1 is generally regarded to function as a “housekeeper”, although the reason and mechanism behind the increased COX-1 expression during late/hypertrophic differentiation remains to be elucidated [7].

Inhibition of COX-2 activity from the start of differentiation specifically decreased expression of hypertrophic markers, while leaving chondrogenic markers unchanged. These findings confirm a specific involvement of COX-2 over COX-1 in chondrocyte hypertrophy [22]. An unanswered question however remains how COX-2 can be specifically involved in regulating chondrocyte hypertrophy over COX-1, while the sole task of both is the synthesis of the PGH_2_ substrate for the synthesis of specific prostaglandins by dedicated downstream enzymes. A possible explanation might be found in the housekeeping function of COX-1 and that inhibiting COX-1 causes deleterious effects on the chondrocyte’s PGH_2_ levels, while PGH_2_ synthesis by COX-2 might occupy only a small additional part of the cellular PGH_2_ pool. Alternatively, a differential involvement of COX-1 and COX-2 in the synthesis of specific prostaglandins could well be possible though, as we found that specific inhibition of COX-1 or COX-2 differently affected the secreted levels of specific prostaglandins in the culture supernatant. COX-1 inhibition showed decreased PGE_2_ concentrations but no effects or even increased concentrations were observed for the other prostaglandins. COX-2 gene expression was found to be upregulated under influence of COX-1 inhibition and might explain the increased prostaglandin levels by redundancy mechanisms, although this would still not explain why PGE_2_ is specifically inhibited by COX-1 inhibition as opposed to the other measured prostaglandins. Inhibition of COX-2 resulted in decreased levels of all the prostaglandins but PGD_2_. Possible cellular rescue of COX expression under inhibitory conditions appears not to be mutually reciprocal, as opposed to above; COX-1 expression did not increase in the COX-2 inhibited conditions and would thus not provide an explanation for the inability for COX-2 inhibitors to down regulate PGD_2_ levels. Presently we do not have an explanation for this observation.

We demonstrated that PGE_2_, PGF_2α_ and in lesser extent also PGD_2_ (in the concentrations tested) have positive effects on chondrogenic and hypertrophic gene expression in ATDC5 cells, whereas addition of TXA_2_ resulted in increased hypertrophic gene expression only. Some of these observations are in line with the report from Jacob *et al*., who described a positive effect on chondrogenic outcome after addition of PGD_2_ and PGF_2α_ [28]. However, PGE_2_ did not increase expression of chondrogenic markers in this report, whereas in our study it resulted in the most pronounced effects from all tested prostaglandins. Differences of culture models; redifferentiating mature articular chondrocytes *versus* differentiating progenitor cells could be responsible for this discrepancy. In line with our results, several studies show that the addition of PGE_2_ result in increased cAMP levels and PKA signalling [15, 29] which led to increased mesenchymal progenitor cell chondrogenesis. Similarly, addition of PGF_2α_ resulted in an increase of chondrogenic marker expression in a rat chondrocyte cell line and mature chondrocytes [29, 30].

Addition of TXA_2_, surprisingly, only increased hypertrophic marker expression. This correlates with the effects of the COX-2 specific inhibitors, which resulted in decreased TXA_2_ expression and specific decreased hypertrophic gene expression. This may point to a role for COX-2 in TXA_2_ production and chondrocyte hypertrophy, which will be subject of further study. Another interesting observation is increased Sox9 expression after PGD_2_ treatment, as it was previously described that PGD_2_ was able to induce Sox9 transcriptional activation and subsequent differentiation of sertolli cells via its adenylcyclase-coupled DP1 receptor induced cAMP-dependent protein kinase A (PKA) signalling, which resulted in phosphorylation and nuclear translocation of Sox9 [31]. Although we investigated a different cell type in our study, one micromolar PGD_2_ resulted in a similarly increased Sox9 expression, which likely explains the subsequently increased Col2a1 expression. However, whether the action of PGD_2_ in chondrocytes is executed by the same signalling cascade needs further investigation.

The herein presented data show that the various prostaglandins synthesized by differentiating ATDC5 progenitor cells exert very different effects on ATDC5 chondrogenic outcome. After verification in chondrogenically differentiating human progenitor cells, these results may challenge the use of various non-steroidal anti-inflammatory drugs (NSAIDs) for progenitor cell-based cartilage regenerative medicine approaches, as slight differences in COX-selectivity between NSAIDs may lead to pronounced and unexpected differences in prostaglandin synthesis, which in turn will differentially affect the chondrogenic outcome of the differentiation process.
